# Lab-on-a-Scalpel: Medical Tool Incorporating a Disposable Fully 3D-Printed Electrochemical Cell Promoting Drop-Volume Chemical Analysis in the Operating Theater

**DOI:** 10.1021/acs.analchem.5c00599

**Published:** 2025-05-12

**Authors:** Anastasios V. Papavasileiou, Lukáš Děkanovský, Zdeněk Sofer

**Affiliations:** Department of Inorganic Chemistry, 52735 University of Chemistry and Technology Prague, Technicka 5, Prague 6 16628, Czech Republic

## Abstract

Surgical operations are intricate and invasive procedures that require continuous monitoring of the patient’s biochemical profile. Point-of-care testing would allow healthcare professionals to identify abnormalities and make the necessary interventions to minimize the risk of complications and ensure patient safety. To this end, we report the development of a disposable and compact fully 3D-printed electrochemical cell incorporated into a medical scalpel (Lab-on-a-Scalpel), aiming to promote on-site (electro)­chemical analysis in the operating theater. This multifunctional device minimizes the number of instruments needed during surgery and can be fabricated on-demand by using a desktop-sized 3D printer at a very low cost. The performance of the Lab-on-a-Scalpel sensing device was evaluated over various electrochemical techniques (cyclic voltammetry, amperometry, and differential pulse voltammetry) and different setups (stirring, drop-volume analysis, polarization potentials, etc.) for the determination of epinephrine. Results showed attractive analytical figures-of-merit, with the limit of detection (LOD) reaching 0.13 μM, and high accuracy in recovery studies conducted on artificial blood samples. Our findings suggest that Lab-on-a-Scalpel is a valuable tool that enables near-patient diagnostics with a minimum sample volume and holds promise to become an essential tool for robotic-assisted surgery.

## Graphical abstract

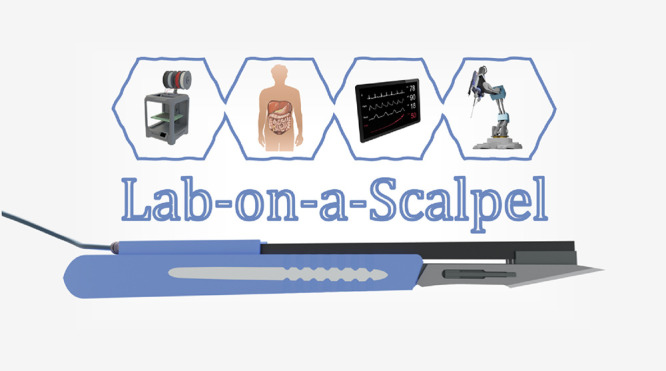

## Introduction

Surgical operations are critical and complex procedures due to their invasive nature that disrupts the physiological functions of the human body. The inherent risks associated with such operations include bleeding, infection, damage to surrounding tissues or organs, and adverse effects on medication or implants. These issues can compromise postsurgical recovery, biological functions, or even the survival of the patients. Therefore, the continuous monitoring of their conditions is imperative in order to identify abnormalities and risks and make early interventions.^1–3^ Beyond the typical vital signs that need to be tracked during surgery (heart rate, blood pressure, respiratory rate, temperature, oxygen level, blood loss, fluid levels, etc.), the abrupt concentration change of biomarkers, metabolites, and stress hormones in the biological fluids of a patient is also indicative of surgical complications and abnormalities.^4–6^

The requirements of instantaneous and on-site chemical analysis in operating rooms cannot be met by the current methods of chemical analysis (such as chromatographic^7^ and spectroscopic techniques^8^) due to high instrumentation and operation costs, lack of portability, and the demand of qualified personnel to obtain reliable results. In recent years, mobile testing and rapid diagnostics through Point-of-Care (PoC) technologies have attracted notable interest from the scientific community and medical professionals. A PoC-compatible method relies on affordable, portable, and user-friendly devices that enable reliable and rapid diagnostics outside of a laboratory setting.^9,10^ Employing PoC methods in the operating theater during a surgical procedure provides real-time patient diagnostics by eliminating the need for formal requests of sample analysis to the hospital’s biochemical laboratories. The long waiting times behind test ordering and receiving lab reports can be significantly decreased. This way the clinician can coordinate care by making faster and more informed decisions and engage in improved treatment plans that lead to an overall better patient postsurgical recovery.^11,12^

Electrochemical sensors possess multiple benefits (portability, affordability, easy miniaturization, high sensitivity, etc.) that render them attractive options for on-site chemical analysis and, thus, PoC diagnostic devices.^13^ The integration of carbon allotropes (graphene, carbon black, and more) in thermoplastic materials unlocked the use of 3D printing technology for the on-demand fabrication of low-cost and sustainable electrochemical sensors. This approach enables the fabrication of advanced electrochemical sensing devices without any design constraints.^14^ The desktop size and affordability of a 3D printer in combination with the fast and easy fabrication process render fused deposition modeling (FDM) an ideal method for electrochemical sensor production.^15^

So far, several studies have utilized FDM 3D printing for the fabrication of electrodes,^15^ cells,^16–18^ and devices^19,20^ for analytical purposes. Even though most of these sensors offer promising analytical features, there are some considerations that need to be addressed to be suitable for near-patient monitoring during surgical operations: (i) to minimize the demand for large sample volumes, which are limited and scarce during a surgical procedure, (ii) to provide a compact sensing platform that will integrate all the necessary components without the need of assembling, and (iii) to be cost-effective for single-use to avoid cross-contamination in a sterile environment such as the operating room.

Herein, we present the fabrication of a disposable and compact fully 3D-printed electrochemical cell incorporated into a medical scalpel, enabling near-patient monitoring of surgical operations through a drop-volume voltammetric analysis, introducing the concept of the Lab-on-a-Scalpel (Scheme 1).

**Scheme 1:**
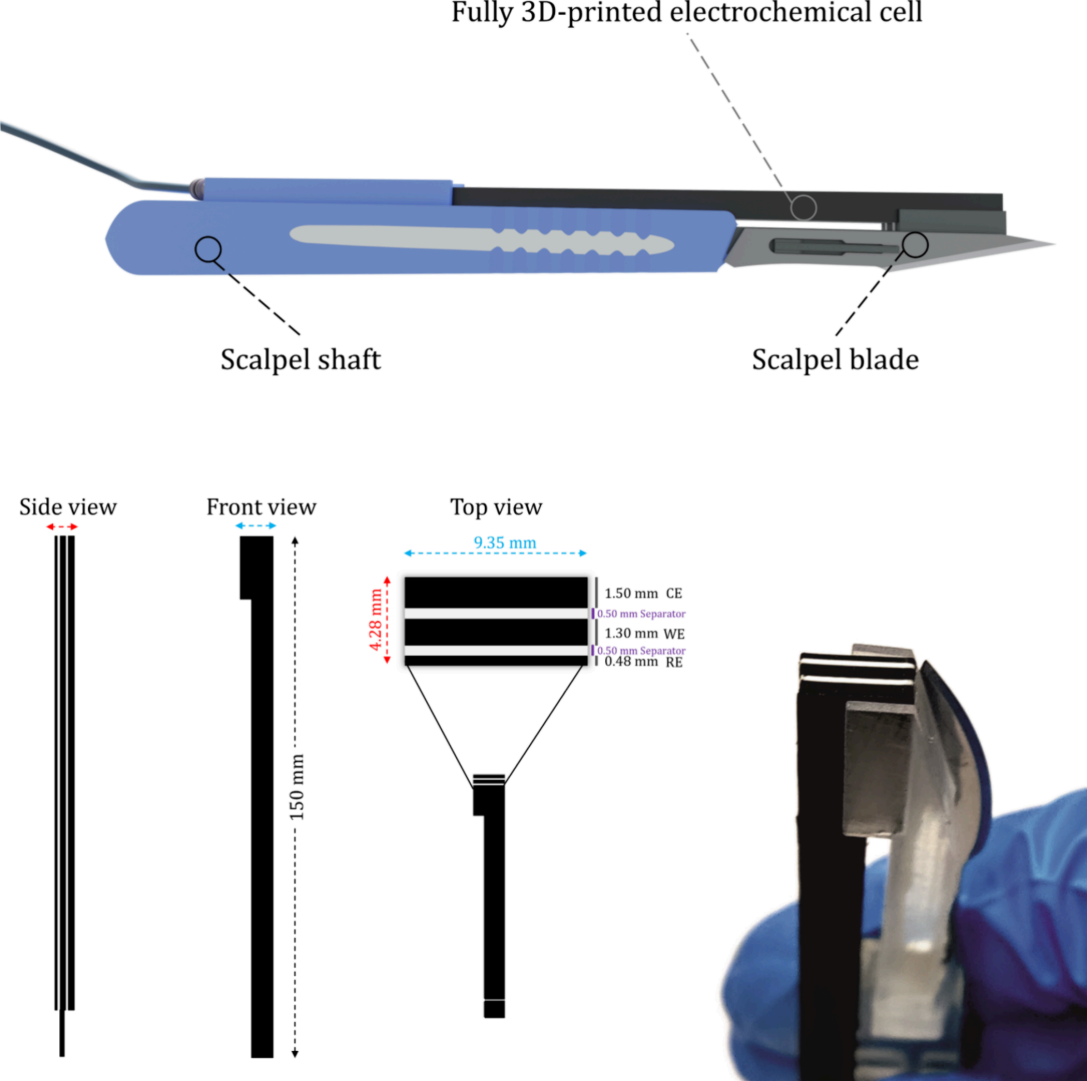
Graphical Illustration of the Lab-on-a-Scalpel Device with the Sensor and the Blade Attached (Top); Side, Front, and Top Views of the Fully 3D-printed Triple Electrode, along with Its Geometrical Characteristics (Bottom Left); and Real Photo of the Lab-on-a-Scalpel Sensing Device (Bottom Right)

Representing a new approach, the Lab-on-a-Scalpel serves as a reliable, fast, and facile sensing platform that can be produced on-demand with a desktop-sized 3D printer at a very low cost (0.40 € per sensor). The combination of an essential surgical tool with an electrochemical sensor results in a multifunctional tool, which limits the number of instruments needed in the operating room. Additionally, it aims to minimize the delays during critical moments in surgery by offering real-time monitoring of the patient’s biochemical profile, contributing to an overall better patient care.

The sensor was investigated for the determination of epinephrine, which is also known as adrenaline. This hormone is crucial as it can be released in humans during stress, while it has also been used by medical professionals to control bleeding, prolong anesthesia, and tackle emergency medical situations such as anaphylaxis or cardiac events.^21,22^ The electrochemical determination of epinephrine using this sensor was explored under different techniques such as cyclic voltammetry (CV), differential pulse voltammetry (DPV), and amperometry, and diverse experimental conditions (drop-volume analysis, polarization potential, stirring, etc.), aiming to fully explore its sensing capabilities across different modes of analysis. The reliability and accuracy of the sensing device were assessed separately for each of the proposed methods of analysis through recovery studies in artificial blood samples.

## Materials and Methods

### Materials

Carbon black/polylactic acid (CB/PLA) filament was obtained from Protopasta (USA). Potassium hexacyanoferrate (III), potassium chloride, sodium dihydrogen phosphate monohydrate, calcium chloride, sodium hydrogen carbonate, d-glucose, and ascorbic acid were purchased from Penta (Czech Republic). Disodium hydrogen phosphate, sodium chloride, and potassium hexacyanoferrate (II) trihydrate were purchased from Lachner (Czech Republic). Epinephrine was purchased from Sigma-Aldrich (USA), and uric acid was purchased from Merck (Czech Republic). Magnesium chloride hexahydrate was supplied by Sigma. The artificial blood solution was prepared by mixing of NaCl 116 mmol L^–1^, NaHCO_3_ 26 mmol L^–1^, Na_2_HPO_4_ 0.9 mmol L^–1^, and NaH_2_PO_4_ 0.2 mmol L^–1^, CaCl_2_ 1.8 mmol L^–1^, KCl 5.4 mmol L^–1^ and MgCl_2_ 0.5 mmol L^–1^,^23^ and phosphate buffer saline (PBS) by mixing Na_2_HPO_4_ (0.1 M) and KCl (0.1 M).

### Fabrication of the Sensor

The scalpel and the triple electrode were designed and sliced using Autodesk Fusion 360 and Prusa Slicer software, respectively. The scalpel shaft was specifically designed to accommodate the 3D-printed triple electrode on one side and the scalpel blades on the opposite side. All components were printed using a Prusa Mini with the following settings: nozzle temperature of 215 °C, bed temperature of 60 °C, printing speed of 50–80 mm/s, printed layer thickness of 0.1 mm, and infill of 100%, with manual change of filament and control of filament contamination by conductivity measurements.

The electrodes of the electrochemical cell were printed with a CB/PLA Protopasta conductive filament, while the nonconductive layers separating them (insulators) and the scalpel shaft were printed using plain polylactic acid (PLA) filament (Figure S1).

The as-printed triple electrode was activated in one-step electrochemical treatment by connecting all three electrodes (working, counter, and pseudoreference) to a single crocodile clip, applying +1.8 V (vs Ag/AgCl) for 900s in 0.1 M PBS (pH 7) with a Pt foil serving as counter electrode.^24^ While the activation is not necessary for counter and preudoreference electrodes, its implementation helps in the homogeneity of the triple electrode surface.

Finally, to avoid water ingress in the counter and pseudoreference electrode and isolate the active area, the triple electrode was coated perimetrically along all the sides—except the top—with a colorless nail polish.^14^

### Printing Process

The fabrication of a fully 3D-printed electrochemical cell can sometimes be a complex procedure due to the need for the integration of two different filament materials in one structure. In particular, CB/PLA and PLA filaments have been used interchangeably for the electrodes and insulators, respectively. The use of a dual-extruder 3D printer that will automatically change the filaments when needed is ideal for a multifilament printing procedure. Such a printing model is able to reduce the fabrication time and produce ready-to-use sensing devices in a single-step printing process. Nevertheless, a dual-extruder 3D printer is an added benefit rather than a necessity. Most commercial 3D printers support multimaterial printing by manual switching of filaments during the printing process, rendering the on-demand fabrication of such sensing devices highly accessible.

### Geometric Characteristics

The sensor was specifically designed with a shape and geometry that allows it to fit and merge within a medical scalpel. It can be easily attached to the rear side, just like the blade, and can be disposed of and replaced on demand (Scheme 1 and Figure S1).

The long base facilitates the easier connection with the potentiostat and at the same time serves as a probe that is able to reach inaccessible body fluids, if needed.

The thickness, the relative position, and the distance between the electrodes were selected based on basic principles of electrochemistry. In this way, the counter electrode has an increased surface area that will not limit the performance of the sensor. At the same time, the working electrode is located between counter and pseudoreference electrodes, with their distance being the shortest possible, yet without being in contact.

The thickness of each one of the material layers determines the length of the sensing interface and thus the surface area influencing the performance of the sensor. Preliminary experiments demonstrated that thicknesses between 0.4 and 2.0 mm in sensors prevail in terms of printability and size effectiveness without any limitations in electrochemical performance. Therefore, a 1.3 mm thickness was selected for the working electrode, and accordingly, the counter electrode and pseudoreference electrode have thicknesses of 1.5 and 0.48 mm, respectively (Scheme 1).

The electrodes were separated by a 0.5 mm insulator printed by plain PLA, allowing minimal spacing between the three electrodes, thus enabling the measurement of a drop-volume solution of 50 μL. This triple electrode design constitutes a compact, simple, and easy-to-prepare approach for a triple electrode. It can serve as a promising alternative to the commercial screen-printed triple electrode, which consist of circular and arc-shaped electrodes that entail a rather complex design and production procedure.

### Structural Characterization

Raman spectra were measured using an inVia Raman spectroscope (Renishaw, UK) in backscattering geometry equipped with a charge-coupled device detector using a DPSS green laser (532 nm, 50 mW), with an applied power of 5 mW and a 20× magnification objective.

### Electrochemical Measurements

Electrochemical measurements were conducted using a Corrtest 4-channel potentiostat at room temperature. Electrochemical characterization was carried out with the electrochemically activated CB/PLA 3D-printed electrode serving as the working electrode, Pt foil serving as the counter electrode, and a Ag/AgCl, 3 M KCl electrode acting as the reference electrode. Electrochemical measurements for the determination of epinephrine were performed with the fully 3D-printed electrochemical cell consisting of electrochemically activated CB/PLA 3D-printed electrodes serving as counter and pseudoreference electrodes, depicted in Scheme 1.

Electrochemical impedance spectroscopy (EIS) was carried out in a frequency range from 100 kHz to 0.1 Hz, with an amplitude of the applied AC potential of 5 mV, superimposed on a DC potential of 0.2 V. Cyclic voltammograms (CV) were recorded in 0.1 M PBS pH 6 at 25 mV s^–1^ unless stated otherwise. Differential pulse (DP) voltammograms of epinephrine were recorded in 0.1 M PBS pH 6 in a potential range between −0.2 and 0.6 V using the following waveform parameters: 0.004 V, amplitude: 0.05 V, modulation time: 0.05 s. Under these conditions, the effective scan rate was 0.008 V s^–1^. Amperometric measurements were conducted in stirred (200 rpm) 0.1 M PBS pH 6 at a polarization potential of +0.5 V, unless stated otherwise.

### Recovery Study

To conduct the recovery experiment, artificial blood sample was spiked with various amounts of epinephrine and analyzed after a 40 times dilution under the following conditions: (i) through amperometry at 0.5 V in a 10 mL electrochemical cell stirred at 200 rpm, (ii) through DPV with a 50 μL volume of working solution, and (iii) through DPV in a 10 mL electrochemical cell prior to a 30 s electroless conditioning step. The addition of the sample was followed by three consecutive additions of 1 or 0.5 μM epinephrine. Then, the final concentration of epinephrine was calculated using the standard addition method.

## Results and Discussion

### Activation Treatment

So far, there are numerous reported activation methods for 3D-printed electrodes that involve chemical treatment,^25^ electrochemical treatment,^24^ laser ablation,^26^ mechanical polishing,^27^ spark discharge,^28^ and others. These techniques have been applied individually or in combination to enhance electrode performance.^29,30^ The selection of the most appropriate activation method is dependent on the intended application. In a sterile environment such as a surgical room, electrochemical treatment and mechanical polishing are more appealing techniques thanks to their inherent simplicity. In contrast, techniques involving chemical treatments with toxic or hazardous reagents are unsuitable due to safety concerns, while those requiring additional instrumentation are less practical in surgical settings.

The three electrodes of the Lab-on-a-Scalpel sensing device were activated through an eco-friendly and highly reproducible electrochemical (EC) treatment by applying +1.8 V in 0.1 M PBS, without the involvement of any hazardous and toxic compounds. All three electrodes that constitute the fully 3D-printed sensor are electrochemically activated simultaneously by connecting them altogether in the same crocodile clip, eliminating the need for their sequential activation.

The Raman spectra in Figure S2 display identical features before and after EC activation. The two characteristic bands of carbon-based materials at 1400 (D band) and 1600 cm^–1^ (G band) exhibit the same intensity ratio (*I*_D_/*I*_G_) of 0.87. This ascertains that no structural defects occur upon EC activation. At the same time, EIS data in Figure S3 shows a significant enhancement in the electrochemical properties of the EC activated CB/PLA 3D-printed electrodes with the charge transfer resistance (Rct) decreasing by 2 orders of magnitude. This activation method has been extensively studied over the morphological, structural, and electrochemical characteristics of the CB/PLA 3D-printed electrodes in a previous work of our group.^24^ It offers enhanced sensing capabilities to the electrodes, providing a uniform electrochemical behavior across their surface. Such activity is achieved without deteriorating the morphology and structure of the electrode, as it exposes carbon black over the PLA layer.

### Electrochemical Characterization

The electrochemical behavior of the working electrode was evaluated by monitoring the electron transfer properties of a standard redox system using a conventional three-electrode system (Ag/AgCl as the reference and Pt foil as the counter). For this purpose, 1 mM hexacyanoferrate (III) in 0.1 M PBS pH 7 through CV was carried out at a scan rate of 50 mV s^–1^. Under the same experimental conditions, the suitability of CB/PLA-based counter and pseudoreference^31^ electrode was investigated. The voltammetric profile of the fully 3D-printed electrochemical cell demonstrated in Figure 1 illustrates an identical shape and peak current with the conventional electrochemical cell, shifted by ca. 0.33 V toward negative potential values.

**Figure 1:**
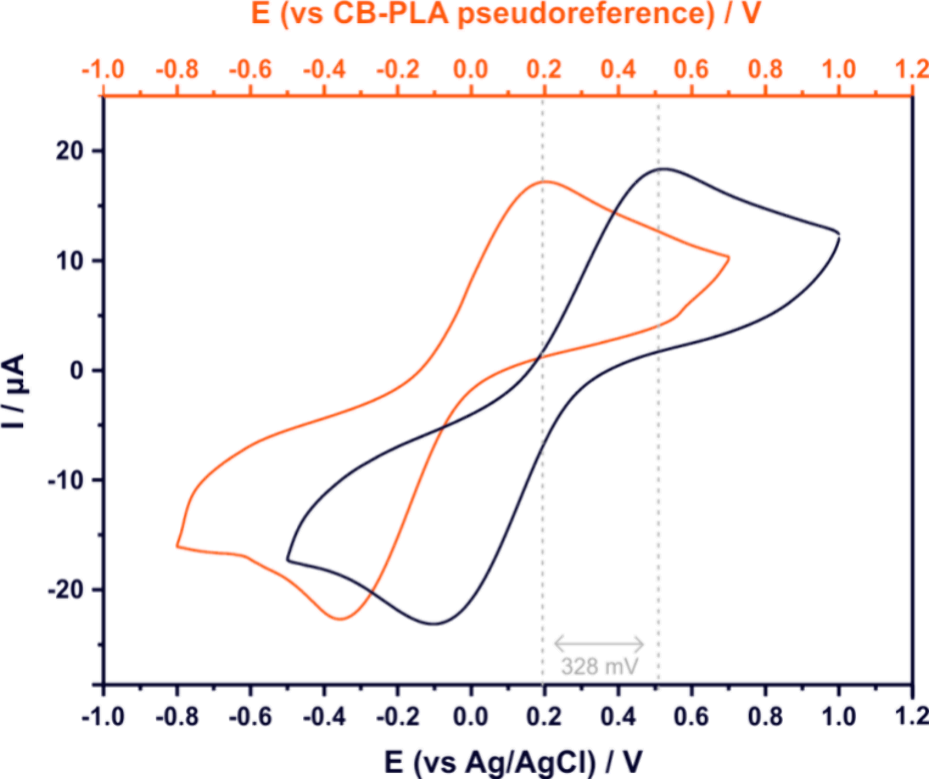
Cyclic voltammograms of CB/PLA 3D-printed electrodes in the presence of 1 mM hexacyanoferrate (III) at a scan rate of 50 mV s^–1^ using a conventional electrochemical cell where Pt foil serves as counter electrode and Ag/AgCl as reference electrode (black); CVs obtained using the fully printed electrochemical cell consisting of a CB/PLA counter electrode and pseudoreference electrode (red).

Additionally, Figure S4 demonstrates the performance of five similarly prepared fully 3D-printed electrochemical cells. The stable peak current response (RSD = 6.15%) ascertains the reproducible behavior of the working electrode, while the constant half-wave potential *E*_1/2_ (RSD = 4.87%) confirms the suitability of the CB/PLA electrode to act as a pseudoreference electrode.

These findings suggest that the fabrication of the fully 3D-printed electrochemical cells is a highly reproducible procedure that yields identical sensing devices that can be used autonomously without the need for any external components (i.e., counter and reference electrode). Henceforth, the fully 3D-printed electrochemical cell is utilized in further investigations.

### Epinephrine Electrooxidation

The sensing capabilities of the fully 3D-printed electrochemical cell were explored with the use of epinephrine as a model analyte. It is undoubtedly one of the most important hormones to track during a surgical operation, as its abrupt concentration fluctuations are indicative of excessive stress in the body functions. Additionally, in many cases, epinephrine is injected into the patient during a surgical operation to minimize bleeding and improve the depth and duration of anesthesia. The lowest effective dose of epinephrine can improve the results of the surgery.^22^ Considering that the accurate and instantaneous determination of epinephrine in the course of a surgical operation is of paramount importance.

Previous studies have shown that epinephrine sensing is favorable in slightly acidic conditions.^32,33^ Data demonstrated in Figure S5 come in accordance with those studies, showing that epinephrine electrooxidation is pH-dependent, and its determination is facilitated at pH 6. Hence, all the subsequent experiments in this research work took place at a pH of 6.

Τhe electrochemical determination of epinephrine relies on its oxidation to the quinone derivative. The mechanism of that reaction was revisited by Bacil et al., who proved that it follows a multistep electron transfer with a swift intramolecular cyclization.^34^ Their findings suggest that the reaction is pH-dependent and highlight that at pH values higher than 5.21 (p*K*_a_), the deprotonated epinephrine can undergo a cyclization after electrochemical oxidation to the quinone derivative. Later on, the cyclic quinone derivative can be electrochemically reduced to form cyclic epinephrine (Figure 2A).

**Figure 2:**
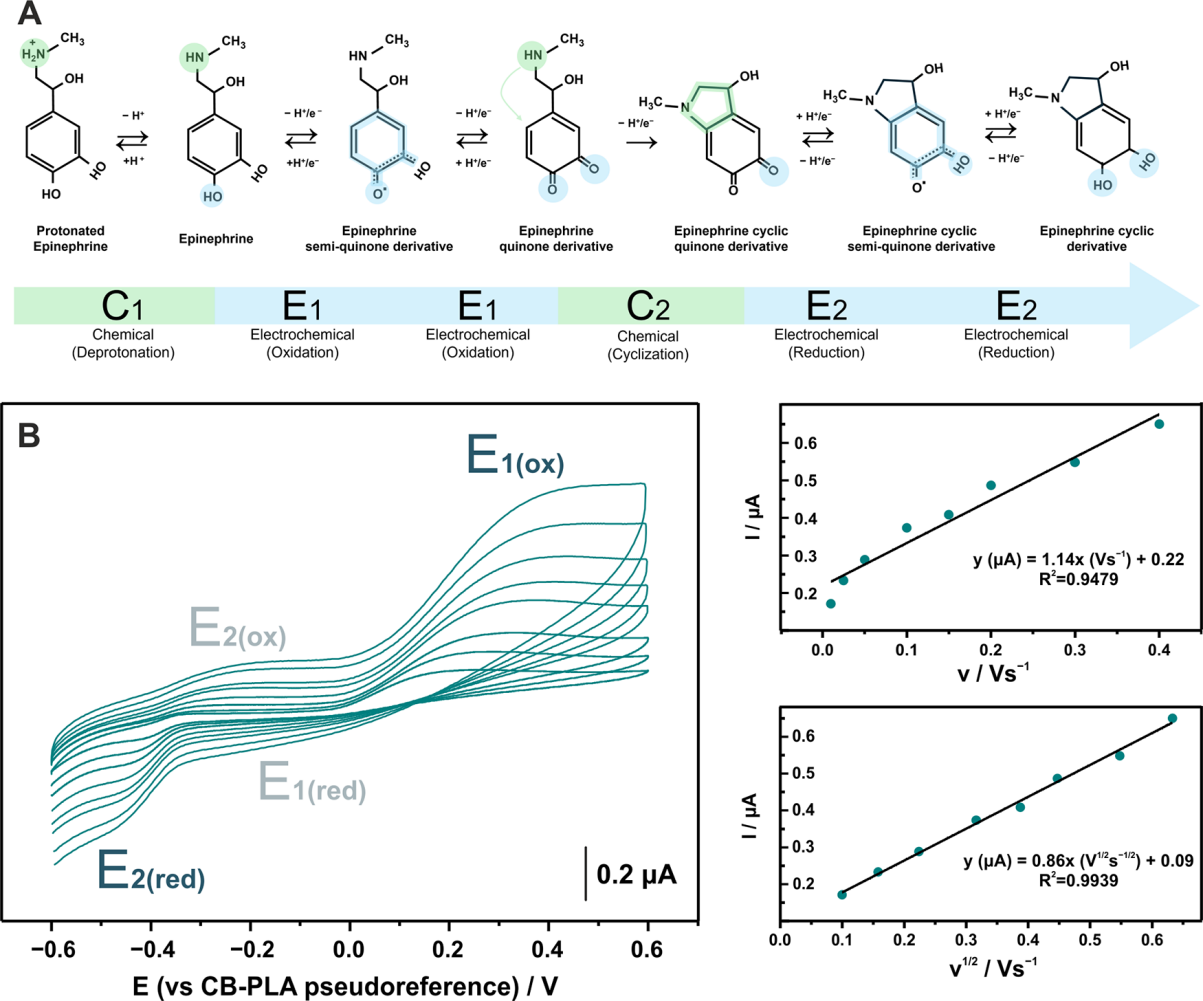
(A) Graphical illustration of epinephrine oxidation reaction following the “C”EECEE mechanism as proposed from Bacil et al.^34^ and (B) Cyclic voltammograms measured with fully 3D-printed electrochemical cell in 0.1 M PBS pH 6 containing 10 μM epinephrine, at scan rates ranging from 10 to 400 mV s^–1^, along with the plot of peak current vs the scan rate and the square root of scan rate with the respective regression equations.

In the interface of the Lab-on-a-Scalpel sensing device (Figure 2B), the electrochemical oxidation of the epinephrine (*E*_1(ox)_) can be clearly identified at an onset potential of +0.2 V (vs CB/PLA), while the electrochemical reduction of the cyclic quinone derivative of epinephrine (*E*_2(red)_) occurs at −0.4 V (vs CB/PLA) at a scan rate of 25 mV s^–1^. Meanwhile, the reverse electrochemical reactions that form the redox couples of those reactions (i.e., *E*_1(red)_ to *E*_1(ox)_ and *E*_2(ox)_ to *E*_2(red)_) are observed only in higher scan rates.

Additionally, the CVs in a wide range of scan rates revealed a linear correlation between the anodic peak current of *E*_1_ and the square root of scan rate (*R* ^2^ = 0.9939), indicating a diffusion-controlled electrochemical process.

### Modes of Determination

The performance of the fully 3D-printed triple electrode for the determination of epinephrine was investigated under different experimental setups by using various electrochemical techniques. Figure 3A–C illustrates the experimental setup corresponding to the techniques: (A) stationary voltammetric mode, (B) hydrodynamic amperometric mode, and (C) differential pulse voltammetry. The figure also highlights key experimental parameters such as whether stirring was applied and/or the volume of the working solution during the analysis. Voltammetric analysis was carried out in 0.1 M PBS (pH 6) in the presence of varying concentrations of epinephrine, and the results are demonstrated in Figure 3A′–C′ along with the corresponding calibration plots (Figure 3A″–C″).

**Figure 3:**
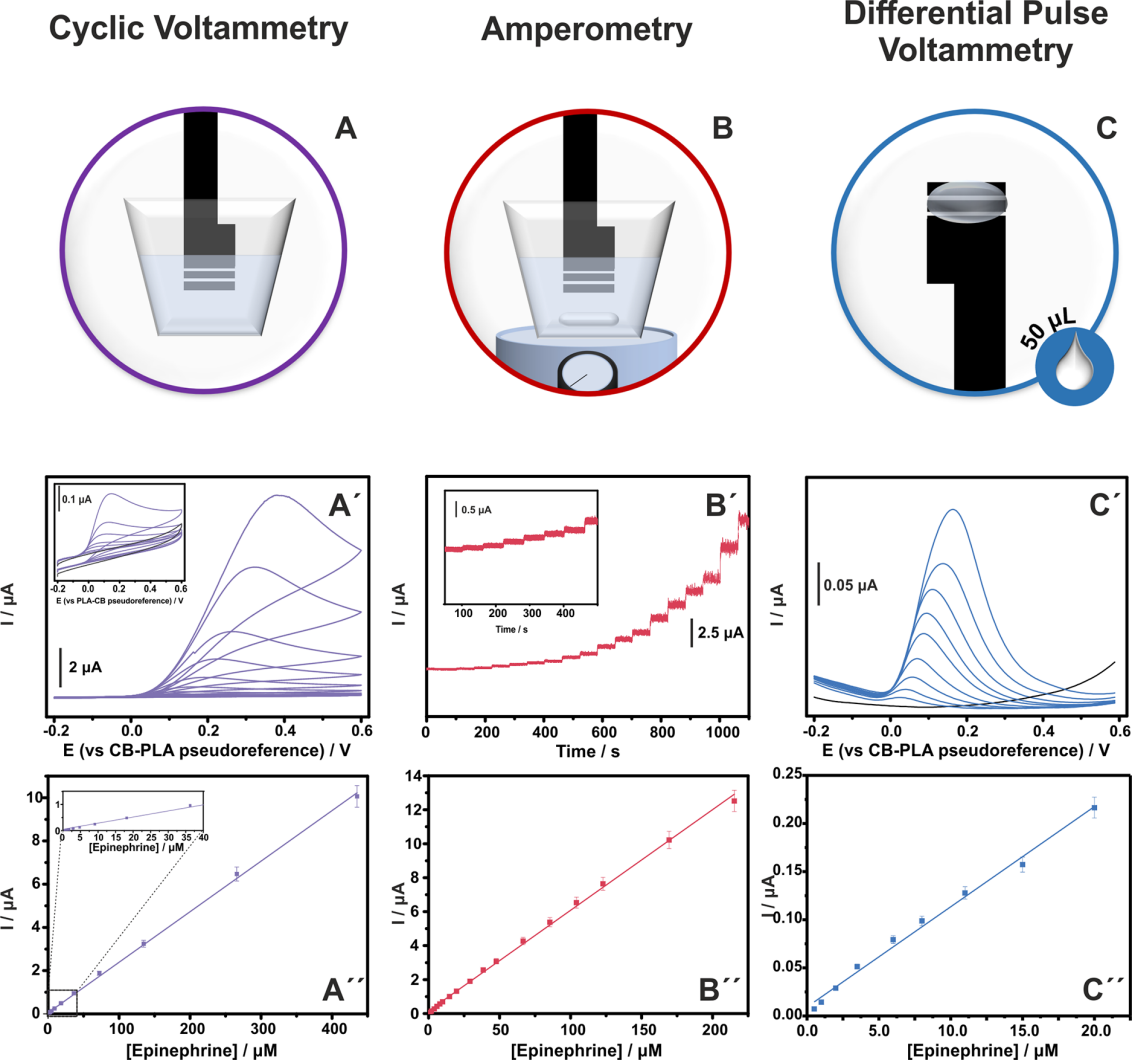
Modes of determination of epinephrine with fully 3D-printed electrochemical cell. (A–C) Graphical illustration of the experimental setup of the measurement, (A′, Α″) CVs in the range from 0.5 to 435 μM of epinephrine at a scan rate of 25 mV s^–1^ and the respective calibration plot, (Β′, B″) amperometric curve at a polarization potential of +0.5 V in a stirred (200 rpm) 0.1 M PBS (pH 6) in the range from 1.0 to 215 μM of epinephrine and the respective calibration plots, and (C′, C″) DP voltammograms of drop-volume analysis in the range from 0.5 to 20 μM of epinephrine and the respective calibration plots.

Cyclic voltammograms performed in a static working solution display a linear dependence between the anodic peak current and epinephrine over the concentration range 0.5–435 μM with the data fitting the equation ip (μA) = (0.0234 ± 0.002) [epinephrine] (μM) + (0.0488 ± 0.0320) (*R* ^2^ = 0.9991). The limit of detection (LOD) was calculated as 3.3σ/s, where σ represents the standard deviation of the intercept of the regression equation and *s* is the slope and found to be 0.14 μM. The calculation was carried out in a narrow concentration range close to the expected LOD rather than the full linear range. Further information regarding the calculation of LOD is presented in the Supporting Information and Table S1 for all the modes of analysis.

The chronoamperometric technique was employed for the continuous monitoring of epinephrine. The amperometric response of a fully 3D-printed electrochemical cell toward five consecutive additions of 10 μM epinephrine at various polarization potentials (Figure S6) reveals a favorable sensitivity emanating from +0.5 V; therefore, it was selected for further exploration. Figure 3B′ demonstrates the amperometric curve of the sensor in a stirred solution containing epinephrine over the concentration range 1.0–215 μM in which the sensor exhibits a linear dynamic range (Figure 3B″); data fit the equation ip (μA) = (0.0593 ± 0.0006) [epinephrine] (μM) + (0.1557 ± 0.0526) (*R* ^2^ = 0.9983), with an LOD of 0.35 μM.

DPV was employed for epinephrine determination through drop-volume analysis. The sensing device was kept vertically, and 50 μL of the working solution was applied, covering the available surface area of the three electrodes (Figure 3C).

The performance of the electrochemical sensor is demonstrated in Figure 3C′ where a peak in an onset potential of 0.0 V (vs CB/PLA) corresponds to the electrooxidation of epinephrine. The anodic peak current exhibits a linear correlation with epinephrine concentration over the range 0.5–20 μM, with the data fitting the equation ip (μA) = (0.0104 ± 0.0003) [epinephrine] (μM) + (0.0094 ± 0.0033) (*R* ^2^ = 0.9927), with an LOD of 0.43 μM.

The CB/PLA pseudoreference electrode is more susceptible to the composition of the working solution, causing a peak shift upon the addition of the analyte. This phenomenon is prevented in the commonly used Ag/AgCl reference electrode thanks to the presence of a fixed concentration of the Ag^+^ and Cl^–^ ions. The extent of peak shifting in CV differs from that in DPV due to the different concentration ranges measured in each case.

### Optimization and Performance

Aiming at enhancing the analytical performance of the sensing device, an electroless conditioning step is introduced by agitating the working solution prior to every measurement so that the measuring species are evenly distributed and interact with the sensing surface. The magnitude of the current response in the presence of 2 μM epinephrine, along with the repeatability of the sensor for 10 consecutive measurements under different conditioning time intervals (0, 30, and 60 s), is assessed.

The repeatability of the sensor under different conditioning time intervals for 10 consecutive measurements displayed in Figure 4A confirms the exceptional stability of the sensor regardless of the conditioning step. The relative standard deviation (%RSD) was calculated as 9.70, 0.91, and 2.41% for 0, 30, and 60 s of conditioning, respectively. Therefore, the suitability of the sensor for conducting a full chemical analysis with a single sensor is deemed more than satisfactory.

**Figure 4:**
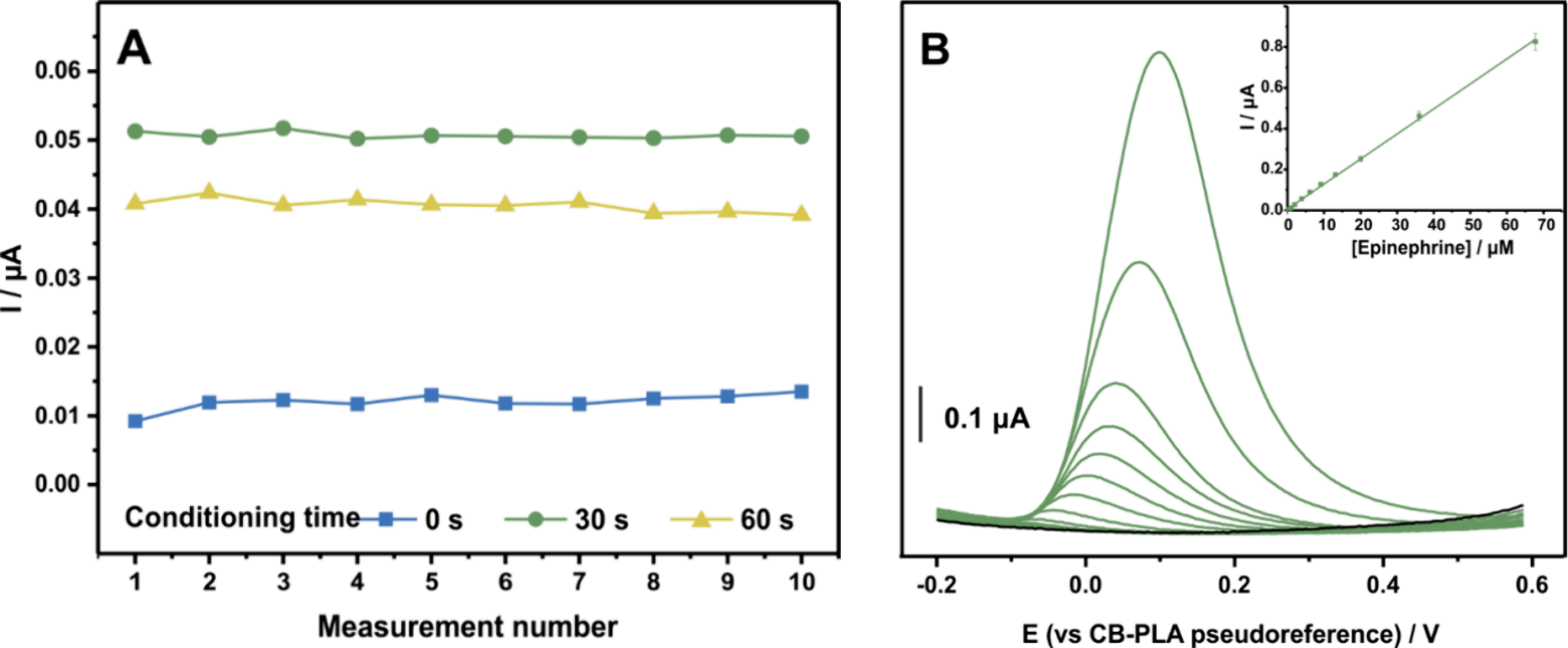
Repeatability of fully 3D-printed in 0.1 M PBS pH 6 containing 2 μM epinephrine for different electroless conditioning steps (0, 30, 60 s) (A) and DP voltammograms over the concentration range of 0.3–68 μM, recorded after 30 s conditioning step (B) with the inset displaying the respective calibration plot.

Moreover, results show that a short conditioning step such as 30 s not only improves the repeatability of the sensor but is also capable of enhancing the current response, leading to a more than 3 times higher sensitivity to the epinephrine determination. These findings suggest that a short conditioning step is helpful yet not obligatory for the determination of epinephrine with the Lab-on-a-Scalpel sensing device.

The performance of the sensor under the optimal experimental conditions is evaluated using DPV. Figure 4B displays the DP voltammograms that were derived after a 30 s conditioning step in the presence of various concentrations of epinephrine in 0.1 M PBS (pH 6). Peak current attributed to epinephrine electrooxidation is linearly correlated with its concentration (Figure 4B inset) in a range of 0.3–68 μM, with the data fitting the equation ip (μA) = (0.0123 ± 0.0001) [epinephrine] (μM) + (0.0063 ± 0.0032) (*R* ^2^ = 0.9988). The LOD was found to be 0.13 μM.

The fully 3D-printed triple electrode after a simple electrochemical activation process and without any modification offers exceptional analytical figures-of-merit for the determination of epinephrine, outperforming the majority of the sensors documented in the literature (Table 1). It allows continuous monitoring and drop-volume analysis of the target analyte, which are coveted and concurrently elusive functions in sensing devices. These features combined with the low-cost and on-demand fabrication render it a top-notch sensing device. Its ability to be incorporated into the rear side of a medical scalpel along with its disposable nature highlights its uniqueness toward near-patient monitoring during a surgical operation.

**Table 1:** Comparison of Reported Electrochemical Sensors for the Determination of Epinephrine with the Current Work^a^

electrode	technique	conditions	linear range, μM	LOD, μM	refs.
mesoporous SiO_2_/CPE	CV	15 s conditioning	1–60	0.6	^32^
SnO_2_/graphene/GCE	SWV		0.5–200	0.017	^35^
C-dots/GCE	DPV		0.05–2.0	0.0061	^36^
niacin/CPE	CV		20.66–174.4	0.0113	^37^
Ti_2_CT_*x*_/GCPE	amperometry	at 1.2 V	0.02–10, 10–100	0.0095	^38^
Ty/MWCNTs/GCE	DPV		0.6–100	0.51	^39^
2-GMA/ITO	DPV		1–60	0.0035	^40^
Laponite clay-modified IPGE	DPV		0.8–10	0.26	^41^
G-PLA	SWV		4–80	0.23	^42^
CNF-AuNPs/GCE	SWV		50–1000	1.7	^33^
Lab-on-a-Scalpel (CB/PLA)	CV		0.5–435	0.14	this work
	amperometry	at 0.5 V	1.0–215	0.35	
	DPV	drop-volume analysis	0.5–20	0.43	
	DPV	30 s conditioning	0.3–68	0.13	

a Key: SiO_2_, silicon dioxide; CPE, carbon paste electrode; SnO_2_, tin oxide; GCE, glassy carbon electrode; C-dots, carbon quantum dots; Ti_2_CT_*x*_, titanium carbide MXene; GCPE, graphite composite paste electrode; Ty, tyrosinase enzyme; MWCNT, multi walled carbon nano tubes; GMA, reduced graphene oxide/Ti_3_C_2_T_*x*_ Mxene; ITO, indium tin oxide; IPGE, inkjet-printed graphene electrode; G-PLA, graphene-polylactic acid; CNF-AuNPs, carbon nanofiber with electrodeposited gold nanoparticles; CB/PLA, carbon black–polylactic acid.

### Selectivity and Recovery Studies

The selectivity of the sensor was evaluated against electroactive interfering species that are commonly present in biological samples, such as glucose, uric acid, and ascorbic acid. Amperometric tests showed that the presence of a 6 times higher concentration of uric acid and a 50 times higher concentration of glucose resulted in a signal less than 10% of the response of epinephrine, indicating no significant interference in its determination. However, ascorbic acid due to its similar electrooxidation potential negatively affects the determination of epinephrine. This interference can be controlled and/or alleviated through (i) a presurgical diet of the patient minimizing the consumption of vitamin C rich foods and drinks or (ii) chemical methods during analysis that promote ascorbic acid degradation.^43^

The reliability and accuracy of the sensor are investigated by recovery studies in spiked artificial blood samples with known concentrations of epinephrine.

The different modes of analysis were examined using the standard addition method, and the results are demonstrated in (i) Figure 5A for the DPV through a drop-volume analysis, (ii) Figure 5B for the DPV through immersion in a working solution after a 30 s electroless conditioning (200 rpm), and (iii) Figure 5C,D for the amperometry at +0.5 V in a 200 rpm stirring solution.

**Figure 5:**
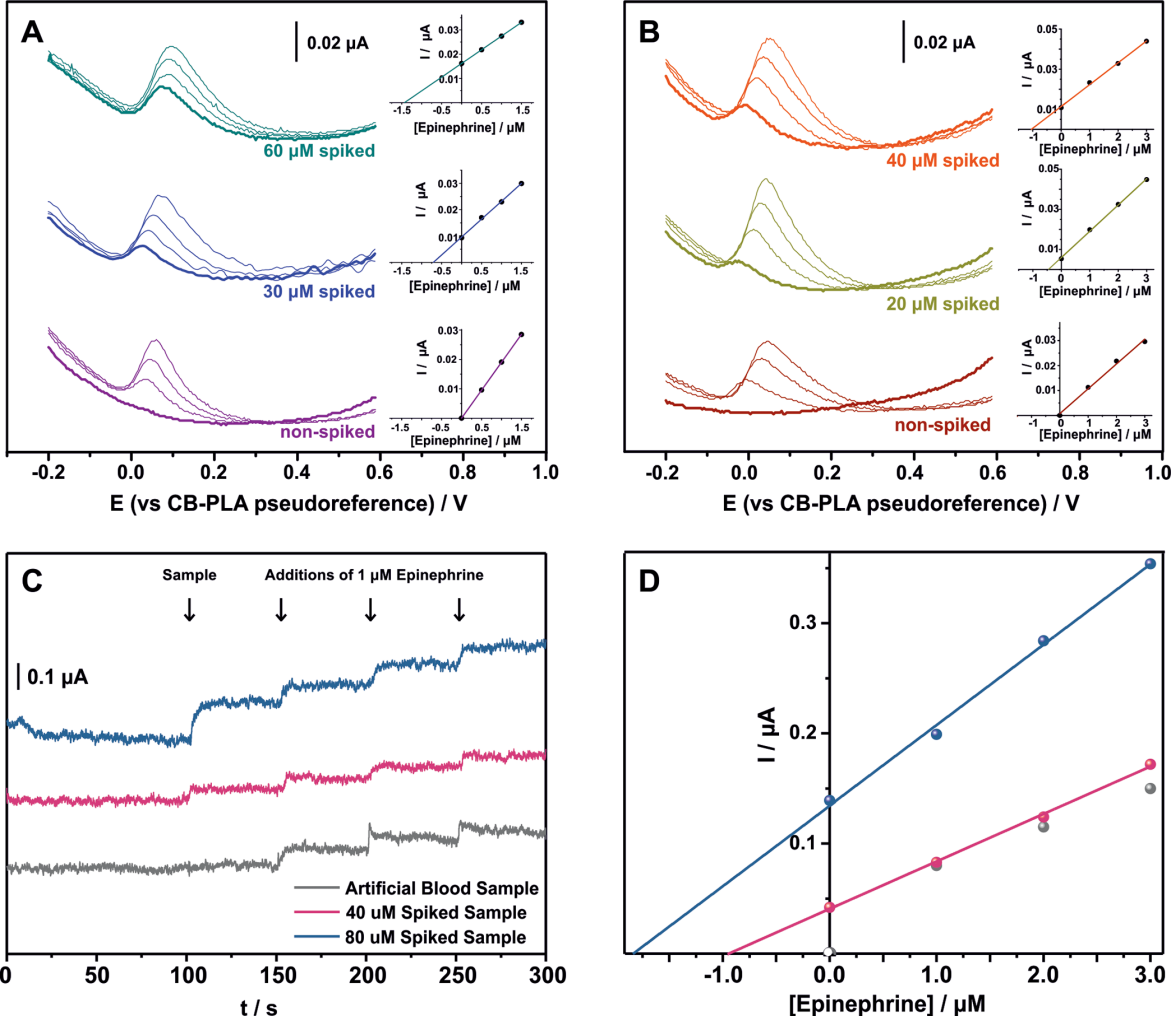
DP Voltammograms of nonspiked and spiked artificial blood samples in 0.1 M PBS (pH 6) along with the respective standard addition plots measured through the drop-volume analysis (A) and through immersing and conditioning (by electroless stirring for 30 s) the fully 3D-printed sensor (B). Amperometric curves recorded with fully 3D-printed sensors at +0.5 V in 0.1 M PBS (pH 6) for the analysis of nonspiked artificial blood samples, spiked with 40 μM and 80 μM epinephrine artificial blood samples, followed by three consecutive additions of 1 μM epinephrine (C) along with the respective standard addition plots (D).

Recovery values ranging between 91 and 105% (Table 2) suggest that the sensing device is capable of operating consistently and accurately across different techniques for the determination of the analyte in an artificial blood sample. These findings underscore the suitability of the sensor for broad applications in clinical diagnostics and biochemical research.

**Table 2:** Determination and Recovery of Epinephrine in an Artificial Blood Sample

technique	conditions	added, μM	found, μM	recovery, %
amperometry	200 rpm stirring	0	N.D.	
		40	37.68	94.20
		80	73.70	92.12
DPV	30 s conditioning	0	N.D.	
		20	18.26	91.31
		40	41.86	104.65
DPV	drop-volume analysis	0	N.D.	
		30	28.48	94.95
		60	57.22	95.37

## Conclusions

In this work, we successfully developed a novel, compact, and disposable fully 3D-printed electrochemical cell incorporated into the rear side of a medical scalpel, named Lab-on-a-Scalpel. It is a multifunctional tool aiming to promote the intraoperative biochemical monitoring of patients through chemical analysis while minimizing the number of instruments required during surgery. The scalability and on-demand fabrication of the sensor along with the very low cost (0.40 € per sensor) render it a viable and practical alternative to conventional analytical methods. This device holds promise to enhance the efficiency and safety of surgical procedures through immediate near-patient diagnostics. Hence, this approach allows healthcare professionals to make timely and more informed decisions and handle more effectively any detected abnormalities.

Lab-on-a-Scalpel was successfully applied in the electroanalytical determination of epinephrine through different electrochemical techniques, showing competitive analytical figures-of-merit without the need for any modification. The high sensitivity (LOD of 130 nM), the outstanding stability (0.91% RSD of repeatability), and the exceptional accuracy (recovery of 91–105%) render Lab-on-a-Scalpel a promising and reliable sensing device, which shows potential for the implementation across a wide range of biochemical analytes. Moreover, its ability to conduct full chemical analysis with just a 50 μL drop is of high importance during a surgical procedure due to the limited availability of the sample.

Even though the development of this sensing device represents a significant step toward chemical analysis in the operating room, further advancements are needed to fully realize its capabilities. Addressing challenges associated with direct contact with biological fluids, such as biofouling and electrode passivation, will be essential for optimizing the device for in situ chemical analysis. The utilization without the need for sample preparation can fully exploit its potential.

All in all, Lab-on-a-Scalpel connects analytical chemistry and biomedical engineering by combining an electroanalytical sensor with a medical tool to construct an advanced multifunctional device. This device can promote point-of-care diagnostics in surgical theaters and holds promise to become an essential tool for robotic-assisted surgery. Its integration in such robotic platforms can improve the accuracy of the procedure by adjusting its actions in response to the biochemical profile of the patient.^44^ In this manner, this can improve the efficiency of the procedure and ensure patient safety. Upon connection with wireless data transmission technologies, Lab-on-a-Scalpel can communicate with other surgical instruments and monitoring systems that will pave the way for its broader adoption in next-generation, smart surgical environments.

## Supplementary Material



## Data Availability

The data sets generated and/or analyzed during the study are accessible via the Zenodo repository: https://zenodo.org/records/14067710.

## Notes

Real photos of the Lab-on-a-Scalpel sensing device and the electrical connection, Raman spectra of CB/PLA 3D-printed electrodes before and after EC activation, bode plots of EIS data of CB/PLA 3D-printed electrodes before and after EC activation, reproducibility of working and pseudoreference electrodes through CV in hexacyanoferrate (III), pH study of the electrooxidation of epinephrine on the CB/PLA electrode interface, calculation of the limit of detection, and optimization of the polarization potential for the amperometric determination of epinephrine (PDF)
