# Supplementary material for: Lab-on-a-Scalpel: Medical Tool Incorporating a Disposable Fully 3D-Printed Electrochemical Cell Promoting Drop-Volume Chemical Analysis in the Operating Theater
Source: Anal Chem. 2025 May 12;97(20):10709–19. doi: 10.1021/acs.analchem.5c00599 (PMC12120817; doi:10.1021/acs.analchem.5c00599)
Supplement: Supplementary file 1 [file ac5c00599_si_001.pdf]

Supporting Information

Lab-on-a-Scalpel: Medical tool incorporating a disposable fully 3D-printed electrochemical cell promoting drop-volume chemical analysis in the operating theater

Anastasios V. Papavasileiou[†]*, *Lukáš Děkanovský*[†], *Zdeněk Sofer*[†]*

[†]Department of Inorganic Chemistry, University of Chemistry and Technology Prague, Technicka 5, 16628 Prague 6, Czech Republic

Table of Content

1. Real images of the Lab-on-a-Scalpel sensing device

2. Raman Spectra of CB/PLA 3D printed electrodes before and after EC activation

3. EIS bode plots of CB/PLA 3D printed electrodes before and after EC activation

4. Reproducibility of the electrochemical cell through CV in hexacyanoferrate (III)

5. pH study of the electrooxidation of epinephrine on the CB/PLA electrode's interface

6. Calculation of the Limit of Detection (LOD)

7. Optimization of the polarization potential for the amperometric determination of epinephrine

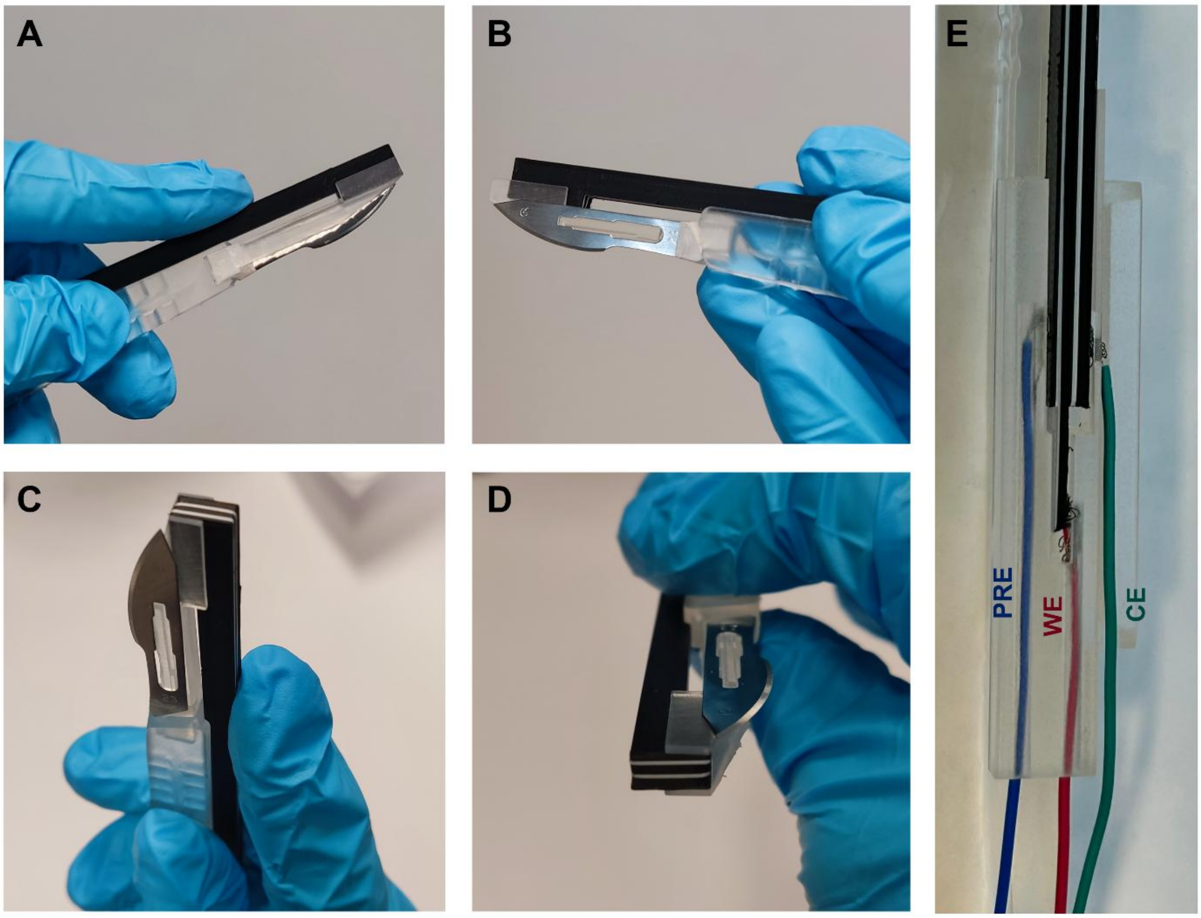

Figure S1. Photos of the Lab-on-a-Scalpel sensing device, focusing on (A, B) the scalpel blade, (C, D) the fully 3D-printed electrochemical sensor and (E) the electrical connection of the electrodes (pseudoreference, working, and counter) to the potentiostat.

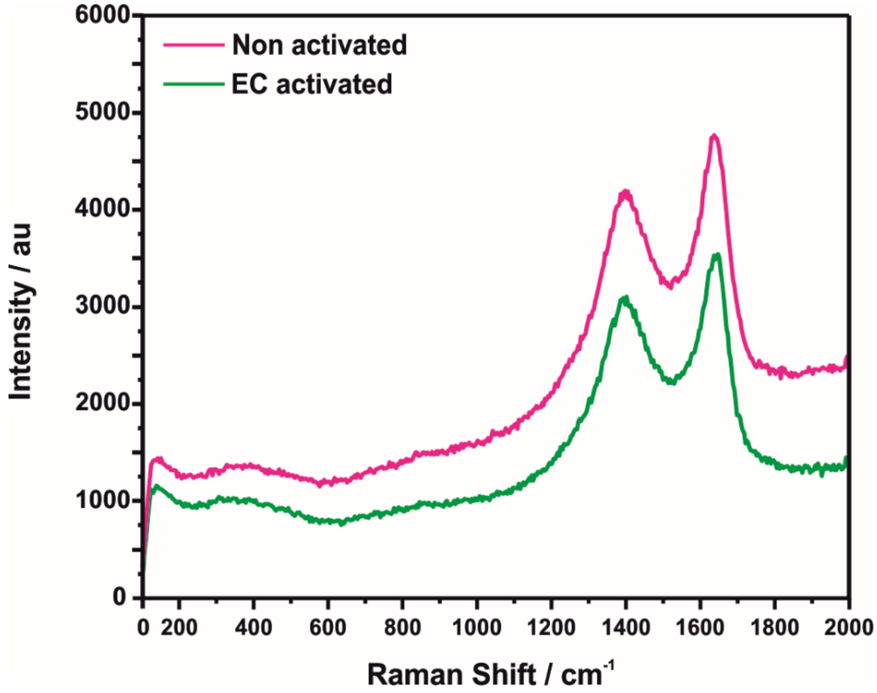

Figure S2. Raman spectra of the CB/PLA 3D printed electrodes before (magenta) and after (green) the electrochemical activation.

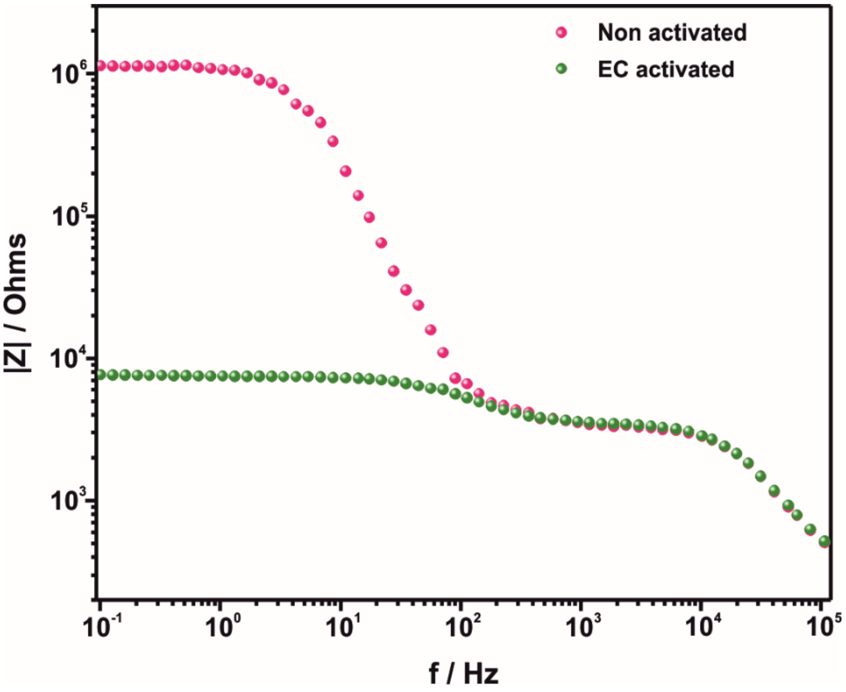

Figure S3. Bode magnitude plots showing the impedance data of CB/PLA 3D printed electrodes in 0.1 M PBS (pH 7) containing 5 + 5 mM hexacyanoferrate (III)/(II) at 0.2 V, before (magenta) and after (green) the electrochemical activation.

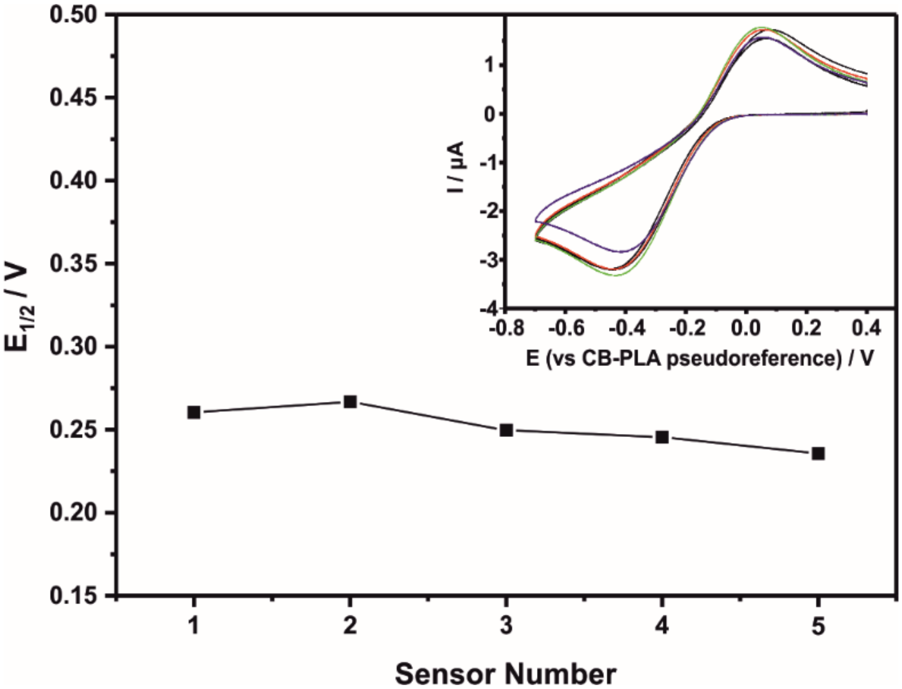

Figure S4 Half wave potential ($E_{1/2}$) over the redox reaction of 0.5 mM hexacyanoferrate (III) of similarly prepared fully 3D-printed electrochemical sensors (N=5) where CB/PLA serves as pseudoreference electrode. Inset graph demonstrates the corresponding cyclic voltammograms recorded with a scan rate of 25 mV s^{-1}

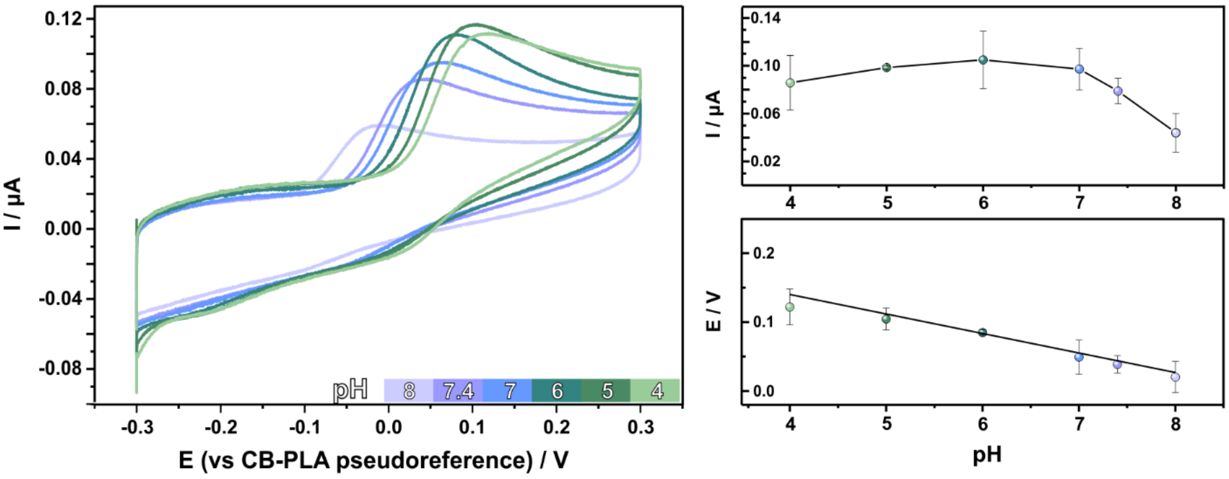

Figure S5. CV voltammograms of fully 3D-printed electrochemical cell in 0.1 M PBS containing 10 µM epinephrine at the pH range 4-8, along with the plots demonstrating the effect of the pH on the anodic peak current (top) and the peak potential (bottom).

Limit of Detection

The Limit of detection (LOD) was calculated using the formula 3.3σ/s where σ is the standard deviation of the intercept of the regression equation and s the slope. To enhance the statistical reliability, the calculation was performed within a narrow concentration range near the expected LOD, rather than across the entire concentration range. Table S1 shows the coefficients of regression equation along with the respective standard deviation both for the entire concentration range and the narrow concentration range utilized for the calculation of LOD.

Table S1. Calculation of Limit of detection (LOD) as 3.3σ/s from the regression equation of the general form: ip (μA)= (a ± SD_a)[Epinephrine] (μM) + (b ± SD_b), where σ = SD_b and s = a

Technique	Range of Concentration / μM	a	SD_a	b	SD_b	R^2	LOD=3.3SD_b/a
CV	0.5 – 435	0.0234	0.0020	0.0488	0.0320	0.9991	
	0.5 - 9	0.0277	0.0003	0.0064	0.0011	0.9995	0.14
Amperometry	1.0 – 215	0.0593	0.0006	0.1557	0.0526	0.9983	
	1 - 20	0.0673	0.0007	0.0109	0.0072	0.9993	0.35
DPV (drop volume)	0.5 – 20	0.0104	0.0003	0.0094	0.0033	0.9927	
	0.5 - 6	0.0132	0.0005	0.0020	0.0017	0.9954	0.43
DPV (30 s agitation)	0.3 – 68	0.0123	0.0001	0.0063	0.0032	0.9988	
	0.3 - 4	0.0157	0.0003	0.0032	0.0006	0.9982	0.13

a, slope; SD_a, standard deviation of the slope; b, intercept; SD_b, standard deviation of the intercept; R^2, correlation coefficient; LOD, limit of detection

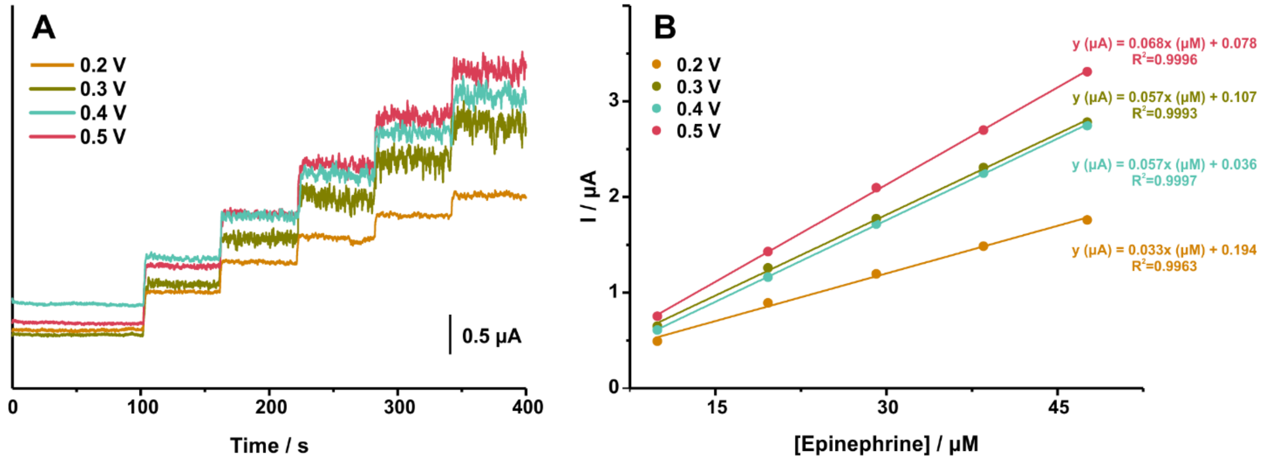

Figure S6. Amperometric curves recorded on sensors at various polarization potentials from +0.2 to +0.5 V in a stirred (200 rpm) 0.1 M PBS (pH 6) electrolyte tracking the response over five consecutive additions of 10 µM of epinephrine (A) along with the respective calibration plots (B).
